# Optical Fiber Pyrometer Designs for Temperature Measurements Depending on Object Size

**DOI:** 10.3390/s21020646

**Published:** 2021-01-19

**Authors:** Arántzazu Núñez-Cascajero, Alberto Tapetado, Salvador Vargas, Carmen Vázquez

**Affiliations:** 1 Electronics Technology Department, Universidad Carlos III de Madrid, 28911 Leganés, Spain; arnunezc@ing.uc3m.es (A.N.-C.); atapetad@ing.uc3m.es (A.T.); 2 Electrical Engineering Faculty, Universidad Tecnológica de Panamá, Ave. Universidad Tecnológica, El Dorado, 0819-07289 Panamá, Panama

**Keywords:** optical fiber pyrometer, temperature measurement, object size effect

## Abstract

The modelling of temperature measurements using optical fiber pyrometers for different hot object sizes with new generalized integration limits is presented. The closed equations for the calculus of the radiated power that is coupled to the optical fiber for two specific scenarios are proposed. Accurate predictions of critical distance for avoiding errors in the optical fiber end location depending on fiber types and object sizes for guiding good designs are reported. A detailed model for estimating errors depending on target size and distance is provided. Two-color fiber pyrometers as a general solution are also discussed.

## 1. Introduction

Temperature measurement techniques can be classified into contact measurements, where thermocouples and resistance thermometers are included, and non-contact techniques, which include infrared (IR) cameras [1,2], pyrometers and micro-Raman spectrometers. Contact temperature sensors present the difficulty of placing them in difficult to access measuring zones; in particular, temperature measurements with thermocouples are highly influenced by the environmental temperature and the thermocouple length [3,4]. Due to the contact, they can also be destroyed when using them in high speed machining [5,6,7,8] and in grinding [9], where the workpiece is moving at a high speed. Some contact sensors present the drawback of a low response time depending on the employed technology and the geometry. Optical fiber sensors (OFS) can be a good solution for working in harsh environments and at high speed [10,11]. Intensity OFS can provide a cost-effective solution but require some self-reference techniques to overcome the negative effect of optical power drifts while measuring [12,13,14]. Apart from that, the high speed rotary movement can destroy the sensing probe when in contact, limiting the sensing capability. These disadvantages can be overcome by using a non-contact self-referenced technique such as optical pyrometers working at two spectral bands in these applications [15,16]. This solution is also interesting when the measuring target is small and the spatial resolution of the measuring technique is important such as in analyzing the evolution of earthquakes [17].

Temperature is an important parameter to be considered in many applications requiring high spatial resolution, for instance, microelectronics [18,19,20], combustion [21,22], electrical discharge machining [23], machining processes [5,6,7,24], laser beam separation [25] and diamond cutting [26]. Typically, in microelectronics, micro-Raman thermography is used for measuring the temperature of devices. In this application, the desired measuring zone is the source-drain gap and a gate with sizes of 4–8 and 0.8–1 μm, respectively [19,20]. For micro-Raman thermography, a laser beam is used for exciting the measuring zone, thus the spatial resolution depends on the laser beam spot. This measurement technique has a long acquisition time, which depends on the material under study and may change from seconds to several minutes [27]. In combustion applications, two-color pyrometers are frequently used because the temperature measurement of the carbon particles, which are in the range of 50 μm [21] to 1.2 mm [22], is not affected by the size of the sensing probe. Another application is the temperature measurement in laser beam cleavage of wafers for controlling the crack propagation and the thermal damage near the cutting zone. In these scenarios, the laser spot is of 0.25 × 0.19 mm^2^ and the difficulty lies on the movement of the laser beam. To overcome this difficulty, two-color optical fiber pyrometers have been proposed for temperature measurement [25]; however, this application requires special optical fibers with large diameters that increases the cost of the measurement equipment. Recent measurements of cutting edge temperature in turning used two-color optical fiber pyrometers placed at distance of 0.3 mm [28] but used wavelength bands that were required to operate with a beam splitter and a filter based on bulk optics with the consequent higher insertion losses and a limited spatial resolution of 535 μm. Another drawback is the time response, which depends on the photodetectors used in the sensing scheme. This also occurs in diamond cutting applications where the cutting zone is determined by the disc width (typically of 70 μm). These measurements are normally done with IR cameras [26] and the difficulty of knowing the target emissivity leads on to an inaccurate absolute temperature.

None of the previous works have proposed a guideline for choosing the right temperature technique based on the target size or the spatial resolution of the technique even in the case of using the two-color optical fiber pyrometer. In this last case, only a brief and tentative, but not a concluding, analysis was shown [29]. Previous works based on two-color optical fiber pyrometry analyzed the behavior without considering the dependence on the distance and, thus, the equations that their authors use despise this effect [5,6,7,30,31,32,33,34]. Nevertheless, it is important to take it into account in a theoretical model to optimize the design parameters of the pyrometer, for instance, the best measuring distance as a function of the target size or the use of a single or dual wavelength band pyrometer. In two-color optical fiber pyrometers, the spatial resolution is related to the optical fiber numerical aperture and the target-fiber distance, allowing a very high spatial resolution when using small core diameters and numerical apertures as shown in [30,35]. It is also important to remember that infrared pyrometry is an important temperature measurement technique, which is still being studied and is employed in defense applications [36] among others.

In this study, we introduce an accurate model describing optical fiber pyrometers for measuring the temperature of objects or localized surfaces with different sizes at high speed. It is important to be able to determine the distance and type of optical fiber, filters and photodetectors to be used when the temperature needs to be measured in localized areas without the need of using different wavelength bands if the surface emissivity is known. It also allows the determining of the distance providing higher optical powers for a specific temperature, thus reducing the requirements on photodetector sensitivity and noise equivalent power. The proposed model of the optical fiber pyrometer response allows the quantifying of temperature errors related to this technique depending on the size of the hot body whose temperature is to be measured. The dependence of the recovered radiation as a function of the object position is also considered. The different cases of the integration limits, depending on the relation between the illuminated surface by the optical fiber versus the target diameter, are discussed. Mathematical equations to model the behavior of optical fiber pyrometers are presented for different targets and fiber diameters. In the simulations, parameters of off-the-shelf devices are considered and, in the case of two-color pyrometer wavelength bands, they are close enough to measure accurate absolute temperatures because of the reduced influence of the emissivity changes of the material on the measuring process [5,7].

## 2. Theoretical Background and Modelling

The base of pyrometry is the measurement of the spectral radiance, *L*, emitted by a target object. The emitted radiance can be mathematically modelled by Planck’s law, as in Equation (1).

(1) $$L\left(\lambda ,T\right)=\frac{C_{1}\varepsilon \left(\lambda ,T\right)}{\lambda^{5}\left(e^{\frac{C_{2}}{\lambda T}}\;-\;1\right)}$$

where *C*_1_ (1.191·10^8^ W·Sr^−1^·μm^4^·m^−2^) and *C*_2_ (1.439·10^4^ μm·K) are the Planck’s radiation constants, *λ* is the wavelength and *T* is the absolute temperature of the body. This equation also considers the emissivity, *ε*, of the object, which represents the effectiveness of the body in emitting energy as thermal radiation.

In two-color optical fiber pyrometers, the radiance of the target is collected by an optical fiber and then the radiation may be split into two wavelength bands. The spectral radiance measured in optical fiber pyrometers is delimited by the acceptance cone of the optical fiber, which depends on the numerical aperture (NA) of the fiber and the locations of the optical fiber and the target surface. In addition, the NA also depends on the fiber refractive index of the core, *n_core_*, and the cladding, *n_cladding_*.

Considering a circular target, *T*, placed perpendicular to the optical fiber, *F*, and whose center is aligned with the axis of the optical fiber, the measured current signal, *I_D_*, of the photodetector for each wavelength band is given by Equation (2).

(2) $$I_{D}\left(T\right)=\int_{\lambda =\lambda_{A}}^{\lambda_{B}}R\left(\lambda \right)IL\left(\lambda \right)\alpha \left(\lambda \right)\int_{S_{T}}\int_{A}L\left(\lambda ,T\right)dA\;dS_{T}\;d\lambda$$

where *λ_A_* and *λ_B_* are the shortest and longest wavelengths of each wavelength band, *R* is the photodetector responsivity, *IL* is the insertion loss of the filter, α is the fiber attenuation coefficient and *dA* and *dS_T_* are the differential elements of the solid angle and the target surface, respectively. 

In a single wavelength band pyrometer only one channel using Equation (2) needs to be considered.

The optical power gathered by the optical fiber is obtained by integrating the radiation projected by each energy emitting differential surfaces, *dS_T_*, into the cone surface projected by the fiber NA. The maximal angle of the acceptance cone, *β_max_*, is defined by:

(3) $$\beta_{\max}=\sin^{-1}\left(\frac{NA}{n_{0}}\right)$$

where *n*_0_ is the refractive index of the medium between the fiber end and the target surface.

The differential elements of power, *dP_dλ,dS__T_*, emitted by each differential surfaces of the target, *dS_T_*, are then projected on the differential elements of the fiber end surface (dF=ududδ). The spectral radiance, *L*(*λ*,*T*), is multiplied by the differential elements of the solid angle, *dA*, to find *dP_dλ,dS__T_*, see Equation (4). The radiation emitted from the target surface depends on the propagation direction and follows Lambert’s Law. Maximum power is obtained when the direction of propagation is at the normal of the plane and decreases with the cosine of the angle, *β*, as shown in Equation (4).

(4) $$dP_{d\lambda ,dS_{T}}=L\left(\lambda ,T\right)\cos\left(\beta \right)dA$$

**Figure 1:**
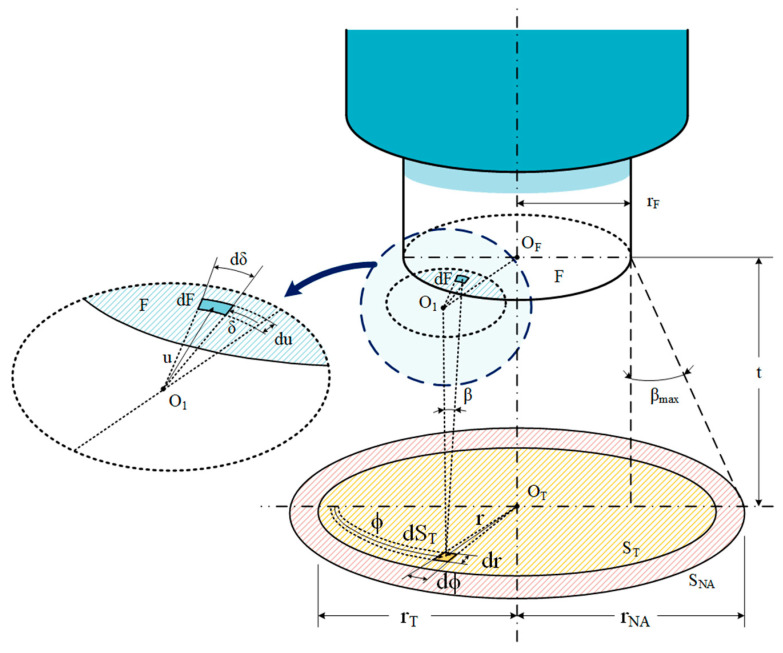
Set up of the target surface, variables and the optical fiber of the pyrometer. Adapted from [37].

The differential element of the solid angle is given by:

(5) $$dA=\frac{\cos(\beta )\;dF}{D^{2}}=\frac{\cos(\beta )\;dF}{t^{2}+u^{2}}$$

where *D* is the radius of the sphere, *t* is the normal distance from the target plane to the fiber surface and *u* is the projection from *dS_T_* to *dF*, see Figure 1. 

Combining Equations (4) and (5) and considering that *cos*(*β*) = *t*/*D*, this leads to Equation (6):

(6) $$dP_{d\lambda ,dS_{T}}=L\left(\lambda ,T\right)\frac{u\;t^{2}}{{\left(t^{2}+u^{2}\right)}^{2}}d\delta du$$

The power coupled to the fiber for rays coming from *dS_T_* with an angle smaller than *β_max_* is calculated integrating Equation (6) over the surface of the fiber core. The result of this mathematical operation is given as follows:

(7) $$P_{d\lambda ,dS_{T}}=\int_{u=u_{min}}^{u_{max}}\int_{\delta =\delta_{min}}^{\delta_{max}}L\left(\lambda ,T\right)\frac{u\;t^{2}}{{\left(t^{2}+\;u^{2}\right)}^{2}}d\delta du$$

**Figure 2:**
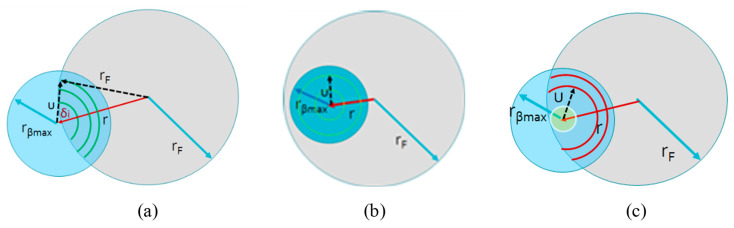
Types of integrations over (**a**) only arcs, (**b**) only circumferences, (**c**) circumferences and arcs.

According to Figure 2, the integration area is defined as the intersection between the fiber core radius, *r_F_*, and the circle of light projected by the *dS_T_* on the fiber surface with radius *r_βmax_*. Both integration limits, *δ* and *u,* depend on the values of *r_F_*, *r_βmax_* and on the separation between the centers of the circles, *r*. Three different scenarios are presented: the area is calculated integrating over arcs Figure 2a, circumferences with a variable radius Figure 2b and a combination of the first and second cases Figure 2c.

In contrast to the work presented in [29], all possible combinations of the integration limits for Equation (7) are described and summarized in Table 1.

**Figure 3:**
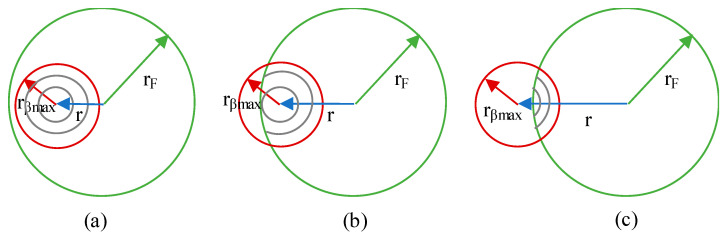
Case I integration sequence as *r* increases (**a**) only circumferences, (**b**) circumferences and arcs, (**c**) only arcs.

**Figure 4:**
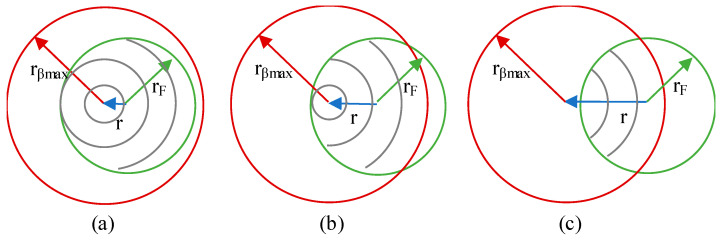
Case II integration sequence as *r* increases (**a**) circumferences and arcs, (**b**) circumferences and arcs with a different upper u limit, (**c**) only arcs.

**Figure 5:**
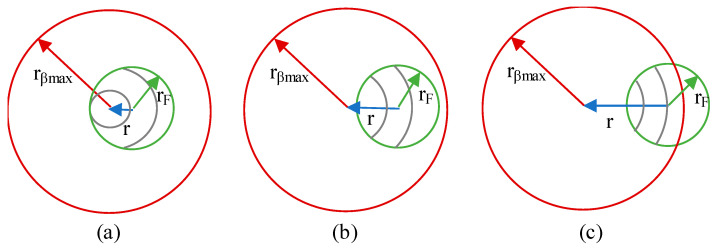
Case III integration (**a**) circumferences and arcs, (**b**) only arcs, (**c**) only arcs with a different upper u limit.

- Integration limit *δ*. In case of integrating over arcs, the limits of *δ* can be calculated using a triangulation method, see Figure 2a. Applying the trigonometric cosine law, the expression of the integration limit of *δ* is given by:
  (8) $$\delta_{max}=-\delta_{min}=\delta_{i}=\cos^{-1}\left(\frac{r^{2}+u^{2}-r_{F}^{2}}{2ru}\right)$$
  In the case of integrating over circumferences, the limits of *δ* are *δ_min_* = 0 and *δ_max_* = 2*π*.
- Integration limit u. The integration limits are defined by the length of the radius and can be changed depending on the ratio between *r_βmax_* and *r_F_*. There are three different scenarios:
  ○ Case I: *r_βmax_* < *r_F_*
    As the radial position, *r*, of the *dS_T_* increases, the integration limits are circumferences, then a combination of circumferences and arcs and, finally, only arcs, see Figure 3.
  ○ Case II: *r_F_* < *r_βmax_* < 2*r_F_*
    As the radial position increases, the integration limits are first a mix of circumferences and arcs, then a mix of circumferences and arcs with different limits and, finally, only arcs, see Figure 4.
  ○ Case III: 2*r_F_* < *r_βmax_*
    As the radial position increases, the integration limits are a mix of circumferences and arcs, then arcs and, finally, only arcs with different limits, see Figure 5.

To compute the total power gathered by the optical fiber, we integrated Equation (7) over all of the target surface. The mathematical expression is given as follows:

(9) $$P_{d\lambda }=\int_{S_{T}}P_{d\lambda ,dS_{T}}dS_{T}=\int_{r=0}^{r_{T}}\int_{\phi =0}^{2\pi }P_{d\lambda ,dS_{T}}rd\phi dr=2\pi \int_{r=0}^{r_{T}}rP_{d\lambda ,dS_{T}}dr$$

where *r_T_* is the target radius and *P_dλ_* is the power per wavelength coupled to the fiber.

Finally, there are two different scenarios in which complex numerical integrations are not required. Therefore, closed equations for the calculus of the power gathered by the optical fiber are found.

- The first scenario was presented in [38] and is given as follows:
  (10) $$P_{d\lambda }=L\left(\lambda ,T\right)\;\pi^{2}r_{F}{}^{2}\left[\frac{1-cos\left(2\beta_{max}\right)}{2}\right]$$
  which is valid when *r_T_* > *r_F_*, specifically for the range given by:
  (11) $$r_{T}>r_{F}+t\;tan\left(\beta_{max}\right)$$

The second scenario, which is described for the first time in this work, is when all of the power emitted for the object at angles less than *β_max_* fall inside the fiber core. This scenario occurs when *r_T_* < *r_F_*, specifically for the range given by:

(12) $$r_{T}<r_{F}-t\;tan\left(\beta_{max}\right)$$

For this case we can use spherical coordinates and integrate the emissivity over the entire surface of the object at angles smaller than the *β_max_* as shown below:

(13) $$P_{d\lambda }=\int_{r=0}^{r_{T}}\int_{\phi =0}^{2\pi }\iint_{A_{s}}^{}L\left(\lambda ,T\right)\;\cos(\beta )\;dA_{s}rd\phi dr=\;\pi \;r_{T}{}^{2}\iint_{A_{s}}^{}L\left(\lambda ,T\right)\;\cos(\beta )\;dA_{s}$$

where the differential element of the solid angle in the spherical coordinates *dA_S_* is given by:

(14) $$dA_{s}\;=\;sin\;\left(\beta \right)\;d\beta \;d\phi_{s}$$

where *β* is the polar angle and *ϕ_s_* is the azimuthal angle. Combining Equations (13) and (14) we found the power gathered by the optical fiber, as follows:

(15) $$P_{d\lambda }=\pi \;r_{T}{}^{2}\int_{\phi_{s}=0}^{2\pi }\int_{\beta =0}^{\beta max}L\left(\lambda ,T\right)\;\cos(\beta )\;sin\;\left(\beta \right)\;d\beta \;d\phi_{s}=\;L\left(\lambda ,T\right)\;\pi^{2}r_{T}{}^{2}\left[\frac{1-cos\left(2\beta_{max}\right)}{2}\right]\;$$

## 3. Simulation Results and Applications

### 3.1. Proposed Model Validation

The first step was to verify and validate the proposed model using the reported data in [29] as a reference. The electro-optical features of the devices used in the simulations are summarized in Table 2 and include a silica glass optical fiber of 100 μm diameter and 0.29 NA, Ge photodetectors and a target with a diameter of 200 μm at a temperature of 2000 °C. The simulations were carried out using a MATLAB script. The main parameters of the simulation are summarized in Table 2. Using a 2 m length optical fiber as reported in [38] and taking the maximum attenuation of the quartz fiber as 12 dB/km at 1.39 μm the light attenuation was 0.02 dB, which was much lower than the filter insertion losses, 0.18 dB. Therefore, along the whole measuring spectral band, the fiber attenuation could be negligible in this simulation.

**Figure 6:**
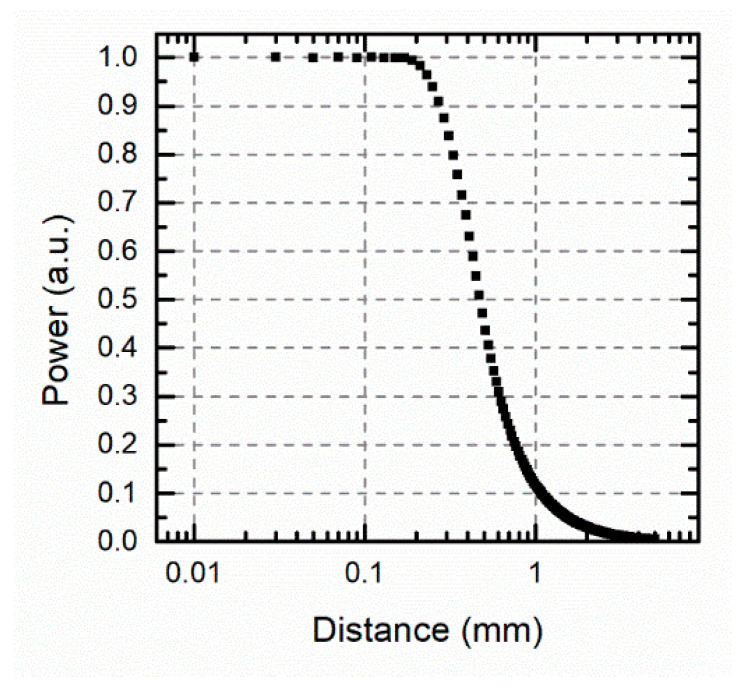
The proposed model simulations with new integration limits. Power measured by the pyrometer for different distances to the target at 2000 °C and using the parameters shown in Table 2.

The simulations depicted that the power incident on the Ge photodetector varied with the distance between the target and the fiber end, see Figure 6. For distances below 0.2 mm, the power was maximum and constant then decreased when the distance increased. The point where the optical power began to decrease is known as *t_c_*, the critical distance between the optical fiber and the target surface, which in this simulation was at the same point as the one reported in [29]. Therefore, the proposed model was verified and validated.

Once the model was verified for a specific case, it was possible to use it with different optical fibers, filters and photodetectors by simply changing the parameters on the code for each instance.

### 3.2. Critical Distance to Target Surface and Spatial Resolution

**Figure 7:**
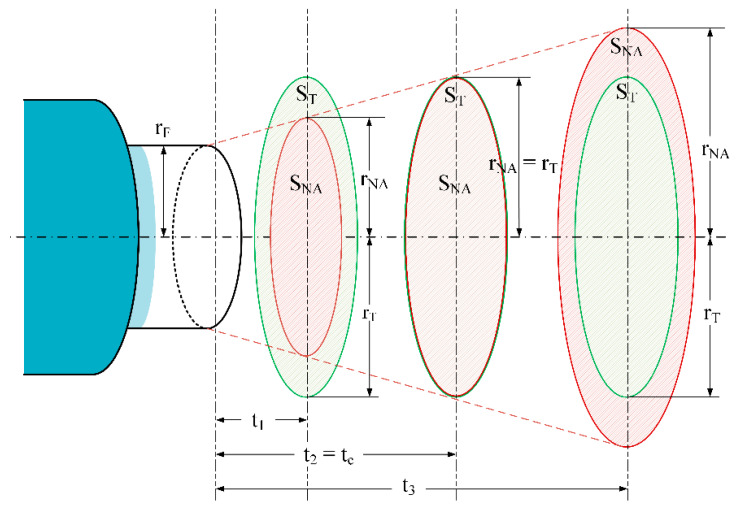
Relations between the numerical aperture radius *r_NA_* and the target radius *r_T_* for different *t* distances.

Figure 7 shows the relation between the numerical aperture radius *r_NA_* and the target radius *r_T_* at different target-fiber distances, *t*. The numerical aperture surface *S_NA_* changed with the target-fiber distance *t* as follows. From the critical distance, *t_c_*, whether *t* < *t_c_*, the target surface *S_T_* was larger than the NA surface *S_NA_* and, in this case, the output power was constant for a specific temperature. As the measuring distance became larger than *t_c_*, the target surface became gradually smaller than the NA surface. In this case, the output power decreased when the measuring distance increased although the temperature was constant.

From a previous analysis, the mathematical expression of the distance *t_c_* is given by:

(16) $$t_{c}=\frac{\left|r_{T}-r_{F}\right|}{\left|\tan\left(\sin^{-1}\left(NA\right)\right)\right|}$$

This means that for any optical fiber pyrometer and depending on the NA surface there is an allowable uncertainty on the optical fiber end position that induces no temperature errors even when using only one channel band. The position of the fiber also affects the spatial resolution of the temperature measurement as reported in [30].

**Figure 8:**
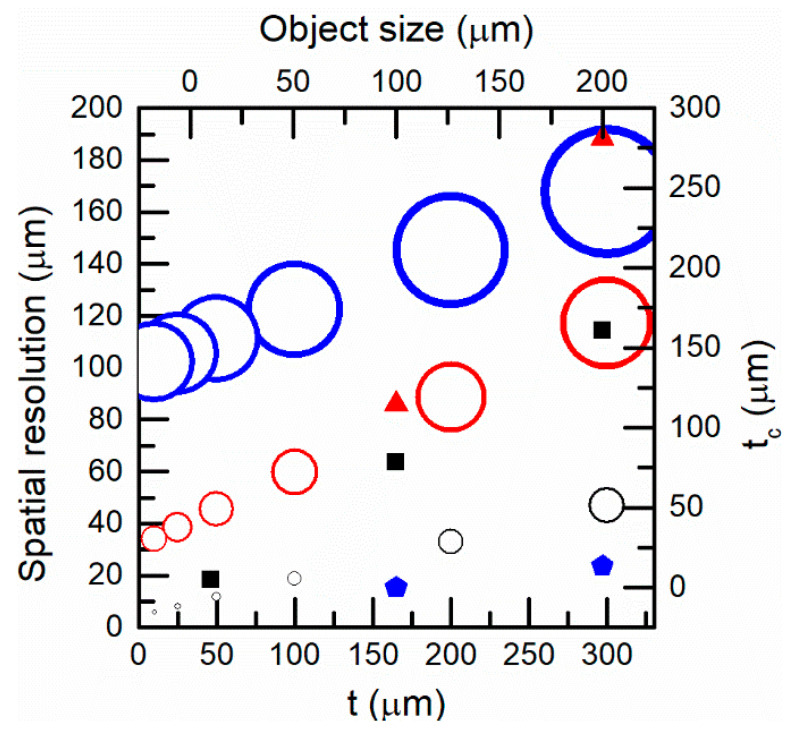
Left y-axis and bottom x-axis: spatial resolution as a function of the target-fiber distance for three different optical fibers (core diameter/NA): black: 9 μm/0.14, red: 62.5μm/0.275, blue: 200 μm/0.2. Right y-axis and top x-axis: *t_c_* as a function of the object size for those fibers: black squares: 9 μm/0.14, red triangles: 62.5 μm/0.275, blue pentagons: 200 μm/0.2.

Figure 8 shows the spatial resolution (defined as the radius of the minimum spot size to be resolved) as a function of the target-fiber distance for different commercial fibers on the left y-axis and the bottom x-axis. The spatial resolution increased when the core diameter of the optical fiber pyrometer was decreased. On the other hand, as the target distance increased, the NA effect reduced the spatial resolution. The right y-axis and the top x-axis of Figure 8 show the value of *t_c_* as a function of the target size for different fibers. For the same optical fiber, the distance *t_c_* increased when the object size increased, see Equation (14). For a resolution of 20 μm with a 9 μm diameter fiber, the optical fiber end should be placed, with high precision, at a 20 μm distance from the target surface. In a fiber comparison, both the core radius and the NA ought to be considered. Thus, for a resolution of 100 μm and a 9 μm/0.14 fiber, the fiber end ought to be placed at 75 μm distance. For a 62.5 μm/0.275 fiber, the fiber end should be placed up to 125 μm. In general, when the object size is similar to the optical fiber size, *t_c_* is very small.

### 3.3. Optical Fiber Pyrometer Designs and Target Size for Avoiding Temperature Errors Using a Single Channel

In the following analysis, the authors used as the reference pyrometer the one presented in [5,7]. It used a multimode fiber with a core and cladding diameter of 62.5/125 μm, respectively, and a numerical aperture of 0.275. An optical filter split the radiation into two spectral bands; one centered at 1.31 μm and the other at 1.55 μm [30]. Two InGaAs photodetectors converted the optical radiation into an electrical signal. The simulations considered a target constant temperature of 1000 °C. In this section, only the 1550 nm channel band was considered. This was equivalent to having a single channel pyrometer with a specific wavelength band from 1460 nm to 1700 nm. 

**Figure 9:**
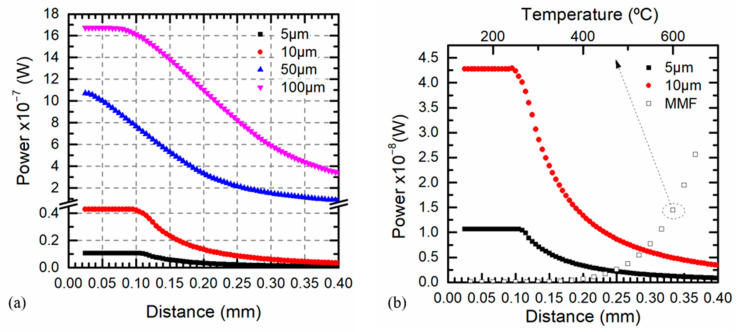
(**a**) Pyrometer optical power at 1550 nm versus the target-fiber distance for different target sizes at 1000 °C (black squares) 5 μm, (red dots) 10 μm, (blue △ triangles) 50 μm, (pink ▽ triangles) 100 μm. (**b**) Pyrometer optical power at 1550 nm versus distance (filled markers) at a constant temperature of 1000 °C for different target sizes and versus temperature (unfilled markers) at a constant distance of 0.3 mm for an infinite surface.

Figure 9a shows the relationship between the pyrometer optical power and the distance for different target sizes ranging from 5 to 100 μm. At a constant temperature according to Equation (14) for target sizes far away from the fiber diameter, *t_c_* was clearly identified and larger distances kept the maximum optical power whose value depended on the target size. On the other hand, if the target size was similar to the fiber diameter, it was difficult to find a constant power for any position. 

Figure 9b shows in the same graph the effect of the distance for different target sizes at a constant temperature of 1000 °C and the effect of the temperature at a constant distance of 0.3 mm for infinite surfaces. Comparing both graphs, a temperature error due to distance misalignments was obtained. For a target size of 10 μm and a target-fiber distance of 0.32 mm, the pyrometer optical power was equal to the power at 500 °C when *r_T_* > *r_F_*. This conclusion meant that the measured temperature would be half of the real one. When the same analysis was performed for a target of 5 μm, the power recovered by the fiber corresponded to a temperature of 575 °C even at close distances. An inaccurate optical fiber positioning led to a reduction of the measured optical power reaching the magnitude of the photodetector noise.

**Figure 10:**
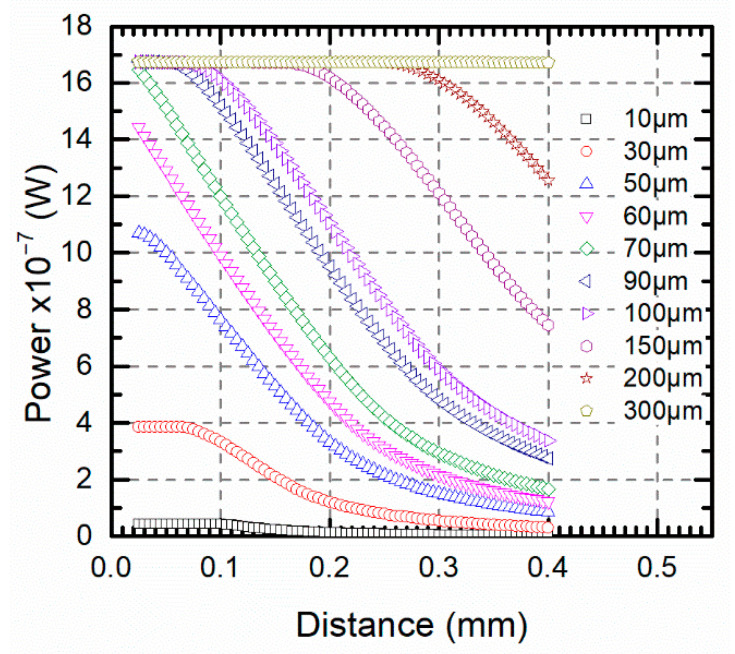
Pyrometer optical power versus the target-fiber distance for different target sizes including *r_T_* > 4*r_F_* in the 1550 nm channel at 1000 °C.

Figure 10 shows the evolution of the optical power for different target sizes at different target-fiber distances. For an optical fiber with a 62.5 μm core diameter and 0.275 NA, the influence of the distance on the optical power was not significant when the target size was large enough in comparison. In such a case, for target sizes of *r_T_* = 300 μm, the optical power was constant for every distance in a range of 0.4 mm. In this scenario, temperature measurements with a single channel pyrometer could be performed with no errors when the emissivity of the material was known. The design parameters of the pyrometer such as the optical fiber diameter and the numerical aperture could be chosen to provide the independence of the measured temperature on the positioning of the fiber when the target size was known. On the contrary, for a specific optical fiber pyrometer, the minimum target size could be estimated providing a constant optical power for a specific temperature for any distance.

**Figure 11:**
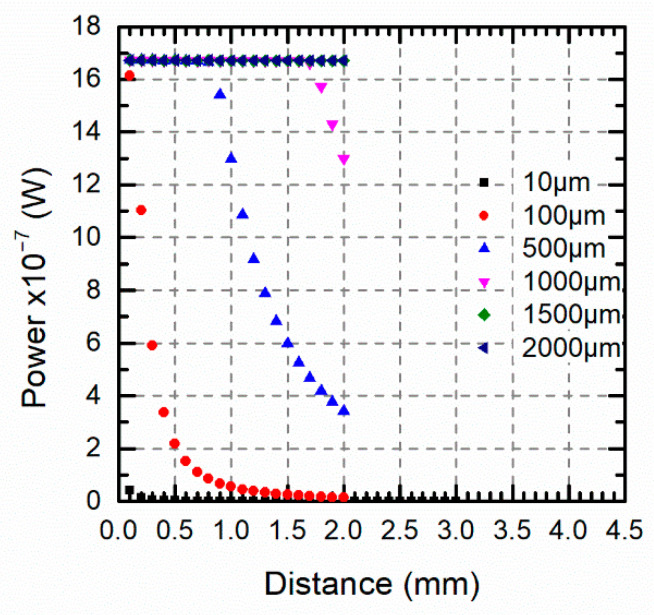
Pyrometer optical power versus the target-fiber distance for different target sizes in a single channel around 1550 nm at 1000 °C.

Using a single channel pyrometer and a target size larger than the optical fiber diameter, but not enough for assuming a distance independent measurement, the model allowed us to estimate the temperature error because of any inaccurate positioning. This statement was only applicable when the emissivity of the target was known. For example, for a 100 μm target, if the target-fiber distance was 1 mm instead of 0.1 mm, the optical power fell up to 1/30 from 1.67 μW to 55 nW, see Figure 11. In this case, the effect of distance disappeared for target sizes greater than 2 mm.

### 3.4. Two-Color Pyrometer for Avoiding Critical Distance Influence on Temperature Errors and Achieving High Spatial Resolution

**Figure 12:**
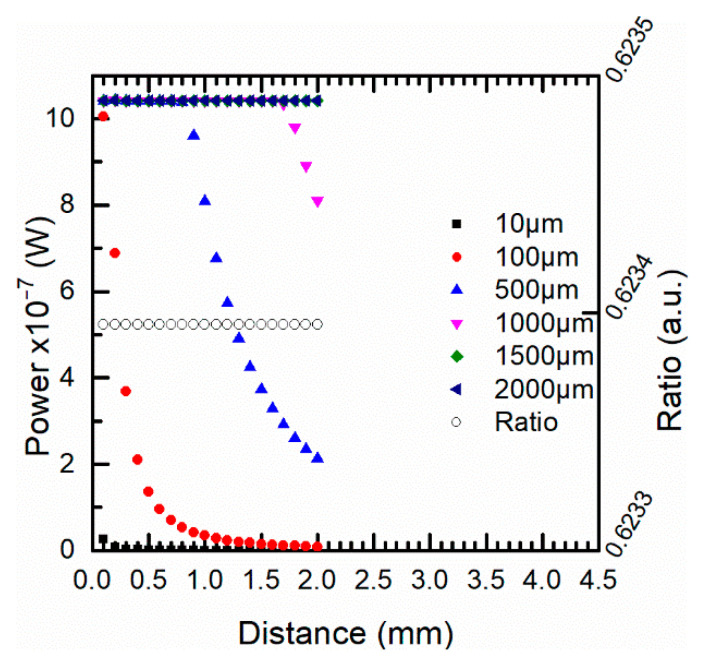
Effect of the target size in the energy recovered at 1000 °C by the 1310 nm channel for different distances. Right y-axis ratio (1310/1550 nm) (unfilled markers) for the different target sizes.

Figure 12 shows the simulations of the optical power at 1310 nm when the target-fiber distance and the target sizes changed from 0 to 4.5 mm and from 10 to 2000 μm, respectively. The behavior of the optical power was similar to the one described for 1550 nm although the energy recovered at this wavelength band was lower. On the other hand, for all target sizes, the power ratio between both channels was independent of the positioning at 1000 °C, see Figure 12.

**Figure 13:**
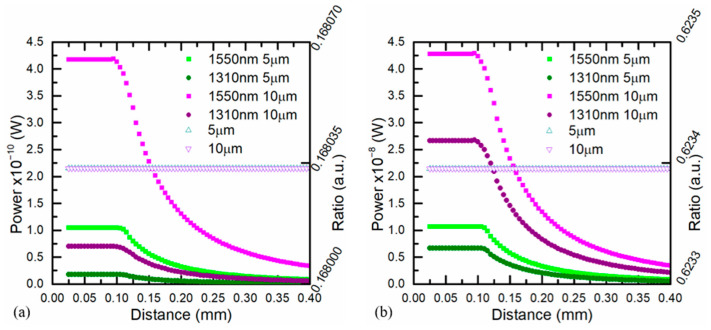
Energy recovered by the optical fiber at both wavelength bands for different target sizes (5 and 10 μm) at (**a**) 500 °C and (**b**) 1000 °C. The right y-axis in both cases shows the ratio (unfilled markers) between both wavelength channels for the different target sizes.

Finally, Figure 13 shows the simulations at both channels for different target sizes and two temperatures of 500 and 1000 °C. The optical power increased for higher temperatures and depended on the distance whether *t* > *t_c_*. Meanwhile the ratio, which was constant for any distance and for all target sizes, was higher when the temperature increased. The use of a two-color pyrometer provided a high performance when the target size was unknown and avoided the effect of distance misalignments. The spatial resolution was not affected by the misalignments.

## 4. Conclusions

A model for simulating the behavior of optical fiber pyrometers for different hot object sizes with new generalized integration limits was shown and validated. Reducing the errors in the measuring temperature using optical fiber pyrometers requires a proper optical fiber end location depending on the object size and optical fiber parameters. Closed equations for the critical distance and the collected power by the optical fiber from the hot body for a different aspect ratio of fiber core and target surface are included. The model calculated the errors related to fiber end misalignments in single channel pyrometers and the design parameters that could reduce this effect. It also validated the optical power ratio independence of distance at a constant temperature for all target sizes in two-color pyrometers. For targets much larger than the optical fiber diameter, there was no influence of the distance target-fiber and an absolute temperature could be measured even for a one-color pyrometer. In the other cases, the model provided guidelines to select the required optical fiber diameter and numerical aperture to use in the pyrometer knowing the target size to be measured for avoiding the positioning dependence.

## Figures and Tables

**Table 1:** Integration limits of *u* according to the ratio between *r_βmax_* and *r_F_* for different values of *r*.

	r				
Case I r_βmax_ < r_F_	0 < r < r_F_ − r_βmax_		r_F_ − r_βmax_ < r < r_F_		r_F_ < r < r_F_ + r_βmax_
	Circumferences		Circumferences	Arcs	Arcs
	u_min_ = 0 u_max_ = r_βmax_		u_min_ = 0 u_max_ = r_F_ − r	u_min_ = r_F_ − r u_max_ = r_βmax_	u_min_ = r − r_F_ u_max_ = r_βmax_
Case II r_F_< r_βmax_ <2r_F_	0 < r < r_βmax_ − r_F_		r_βmax_ − r_F_ < r < r_F_		r_F_ < r < r_F_ + r_βmax_
	Circumferences	Arcs	Circumferences	Arcs	Arcs
	u_min_ = 0 u_max_ = r_F_ − r	u_min_ = r_F_ − r u_max_ = r_F_ + r	u_min_ = 0 u_max_ = r_F_ − r	u_min_ = r_F_ − r u_max_ = r_βmax_	u_min_ = r − r_F_ u_max_ = r_βmax_
Case III 2r_F_ < r_βmax_	0 < r < r_F_		r_F_ < r < r_βmax_ − r_F_		r_βmax_ − r_F_ < r < r_F_ + r_βmax_
	Circumferences	Arcs	Arcs		Arcs
	u_min_ = 0 u_max_ = r_F_ − r	u_min_ = r_F_ − r u_max_ = r_F_ + r	u_min_ = r − r_F_ u_max_ = r + r_F_		u_min_ = r − r_F_ u_max_ = r_βmax_

**Table 2:** Simulation parameters used for the model validation [29].

Optical Fiber Material	Silica glass
Photodetector material	Ge
Responsivity (A/W) @ 1540 nm	0.846
Wavelength band (nm)	800–1700
Numerical aperture (NA)	0.29
Core diameter (μm)	100
Target diameter (μm)	200
Temperature (°C)	2000
Target emissivity	1
Filter insertion loss (dB)	0.18

## Data Availability

Not applicable.
